# Change of Air, or the Pursuit of Health; a Tour through France, Switzerland, and Italy

**Published:** 1831-04-01

**Authors:** 


### XVII.

CHANGE OF AIR, OR THE PURSUIT OF HEALTH; AN AUTUMNAL EXCURSION THROUGH FRANCE, SWITZERLAND, AND ITALY, IN THE YEAR 1829; WITH OBSERVATIONS AND REFLECTIONS ON THE MORAL, PHYSICAL, AND MEDICINAL INFLUENCE OF TRAVELLING-EXERCISE, CHANGE OF SCENE, FOREIGN SKIES, AND VOLUNTARY EXPATRIATION. By *James Johnson*, M.D. Physician Extraordinary to the King. Octavo, pp. 275. Highley, 1831.

IT is hardly necessary to say that no *review* of the above work can be given in this Journal; but a few extracts may be fairly laid before our readers, to enable them to comprehend something of the nature of the performance. The short preface will best explain the design, while it is from the extracts that an opinion can be grounded as to the execution.

PREFACE.

"As the title-page fully expresses the nature of this little volume, a few words only of Preface will be necessary. The Work consists of three Parts, united by the thread of the subject. The FIRST contains some observations on that WEAR and TEAR of mind and body, which we particularly remark in civilized life, and especially in large cities; together with some suggestions as to the antidote or remedy. The SECOND Part consists of reflections and observations made during an excursion through France, Switzerland, and Italy, in the year 1829; partly for recreation—but principally for renovation of health. The THIRD division contains some remarks and speculations on the moral, physical, and medicinal influence of foreign, and especially of an Italian climate and residence, in sickness and in health. In each of these divisions, the author hopes that he has been able to combine utility with some portion of amusement, for those, (and they are many) who, like himself, seek an occasional renovation of health, in a temporary relaxation from the toils and cares of avocation.

Novelty in *description* is now quite out of the question—and from *description* he has generally abstained. Impressions and reflections will continue to be varied, till the minds and features of human beings become similar—and in this respect only, can novelty, or rather variety of sentiment be expected. The construction of this volume will show that the author estimated its value at a very low rate, and consequently imposed but a very moderate tax on the public. He did not—indeed he could not, travel as an Antiquarian, Painter, Architect, Botanist, Geologist, or Politician. He roamed from place to place, as a philosophic observer. It is well known that many people migrate annually to Italy, in search of health—and there find a grave;—while a still larger class go thither in quest of pleasure or improvement, and bring back the seeds of disease. The observations of a medical traveller, not inexperienced in the investigation of climatorial influence on the human constitution, mental and corporeal, may prove useful to those who wander or sojourn on the classic soil of Italy, for any of the above purposes;—while the reflections which he has hazarded on the *moral* effects of foreign residence, or rather *expatriation*, may possibly interest a still wider circle of readers.

The author is well aware of the many imperfections of this little volume; which the nature of his avocations, after the bustle of travelling, gave him little time to correct. But he throws himself on the mercy of the critic and of the reader, with a just confidence that they will make allowance for the circumstances under which the work was composed."

### EXTRACTS.

### I. WEAR AND TEAR.

"There is a condition or state of body and mind, intermediate between that of sickness and health, but much nearer the former than the latter, to which I am unable to give a satisfactory name. It is daily and hourly felt by tens of thousands in this metropolis, and throughout the empire; but I do not know that it has ever been described. It is not curable by physic, though I apprehend that it makes much work for the doctors ultimately, if not for the undertakers. It is that WEAR AND TEAR of the living machine, mental and corporeal, which results from over-strenuous labour or exertion of the intellectual faculties, rather than of the corporeal powers, conducted in anxiety of mind and in bad air. It bears some analogy to the state of a ship, which, though still sea-worthy, exhibits the effects of a tempestuous voyage, and indicates the propriety of re-caulking the seams and over-hauling the rigging. It might be compared to the condition of the wheels of a carriage, when the tyres begin to moderate their close embrace of the wood-work and require turning. Lastly, it bears no very remote similitude to the strings of a harp, when they get relaxed by a long series of vibrations, and demand bracing up.

This WEAR-AND-TEAR COMPLAINT (if the designation be allowed) is almost peculiar to England, and is probably a descendant of the old 'ENGLISH MALADY,' about which so much was written a century ago. And why should it predominate in London so much more than in Paris? The reason is obvious:—In London, business is almost the only pleasure—in Paris, pleasure is almost the only business. In fact, the same cause which produces the WEAR-AND-TEAR malady, namely, hard work, or rather over-exertion, is that which makes our fields better cultivated, our houses better furnished, our villas more numerous, our cottons and our cutlery better manufactured, our machinery more effective, our merchants more rich, and our taxes more heavy than in France or Italy. If we compare the Boulevards, the cafés, the jardins, the promenades of Paris, with corresponding situations in and around the British Metropolis, we shall be forced to acknowledge that it is nearly 'all work and no play' with JOHN BULL during six days in the week, and vice versâ with his Gallic neighbours. Does this 'wear and tear' tell at last upon John's constitution, intellectual and corporeal? I do not speak of the mere labour of the body. The fatigue induced by the hardest day's toil may be dissipated by 'tired Nature's sweet restorer, balmy sleep;'—but not so the fatigue of the mind! Thought and care cannot be discontinued or cast off when we please, like exercise. The head may be laid on the pillow, but a chaos of ideas will infest the over-worked brain, and either prevent our slumbers, or render them a series of feverish, tumultuous, or distressing dreams, from which we rise more languid than when we lie down!" 4.

### II. CARE-WORN COUNTENANCE.

"Whether the *seat* of our feelings and our passions be in the head or in the heart, one thing is certain, that their *expression* is in the countenance. To mask or conceal this expression is the boast of the villain—the policy of the courtier—the pride of the philosopher—and the endeavour of every one. It may appear remarkable that it is much easier to veil the more fierce and turbulent passions of our nature, as anger, hatred, jealousy, revenge, &c. than the more feeble and passive emotions of the soul, as grief, anxiety, and the various forms of CARE. The reason, however, is obvious. Vivid excitement, and tempestuous feeling cannot last long, without destroying the corporeal fabric. They are only momentary gusts of passion, from the effects of which the mind and the body are soon relieved. But the less obtrusive emotions resulting from the thousand forms of solicitude, sorrow, and vexation growing out of civilized life, sink deep into the soul, sap its energies, and stamp their melancholy seal on the countenance, in characters which can neither be prevented nor effaced by any exertion or ingenuity of the mind! The tornado, and the cataract from the clouds, wear not such deep furrows in the mountain's rocky side, as the faintly murmuring rill, whose imperceptible but perpetual attrition effectuates more in the end, than the impetuous but transitory rush of the roaring torrent engendered by the storm, not fed by the spring.

Gutta cavat lapidem non vi sed sæpe cadendo.

This care-worn countenance, in short, is a more obvious mark of the WEAR AND TEAR of mind, in modern civilized life, than premature age:—for age is relative, and its anticipated advance can only be appreciated by a knowledge of its real amount, which can seldom be attained." 8.

### III. ETIOLATION, OR BLANCHING.

"The inhabitants of a city may easily be distinguished from those of the country, by the pallor of their complexions. The care-worn countenance, last alluded to, is generally "sicklied o'er with the pale cast of thought," but the etiolation or blanching which I am now to notice, takes place independently of much thinking or mental anxiety. It cannot, in fact, boast of such an intellectual origin as the other. It is the result of physical, rather than of moral causes —more especially of bad air, inexposure to the light of Heaven, sedentary avocations, inactivity, late hours, &c. I have used the word etiolation, because I think it perfectly appropriate. When a gardener wishes to etiolate, that is, to blanch, soften, and render juicy a vegetable, as lettuce, celery, &c. he binds the leaves together, so that the light may have as little access as possible to their surfaces. In like manner, if we wish to etiolate men and women, we have only to congregate them in cities, where they are pretty securely kept out of the sun, and where they become as white, tender, and watery as the finest celery. For the more exquisite specimens of this human etiolation, we must survey the inhabitants of mines, dungeons, and other subterranean abodes—and for complete contrasts to these we have only to examine the complexions of stage-coachmen, shepherds, and the sailor 'on the high and giddy mast.' Modern Babylon furnishes us with all the intermediate shades of etiolation, from the 'green and yellow melancholy' of the BAZAAR MAIDEN, who occupies somewhat less space in her daily avocations and exercise, than she will ultimately do in her quiet and everlasting abode, to the languishing, listless, lifeless ALBINOS of the boudoir, etiolated in HOTHOUSES, by the aid of 'motley-routs and midnight madrigals,' from which the light as well as the air of Heaven is carefully excluded! Thus penury and wealth, obscurity and splendour, industry and idleness, the indulgence of pleasure and the endurance of pain, all meet at the same point, and, by the mysterious workings of an over-ruling Providence, come to the same level, in this respect, at last! That voluntary dissipation should suffer all the evils attendant on necessary and unavoidable avocation, no one can regret:—but that useful toil and meritorious exertion should participate, and more than participate in the miseries which follow in the train of the 'gay licentious proud,' is a melancholy reflection. The longer we live in this world, however, and the more narrowly we watch the ways and the fate of man, the more we shall be convinced that vice does *not* triumph here below—that pleasure is invariably pursued by pain—that riches and penury incur nearly the same degree and kind of taxation—and that the human frame is as much enfeebled by idleness as it is exhausted by labour." 10.

### IV. LA BELLE FRANCE.

"Of all the countries which these eyes have yet beheld—

——————— A gadibus usque
Auroram et gangem—

LA BELLE FRANCE is the most uninteresting. The flowers—nay even the flatness of Holland—with all its smooth canals and shaded dykes (those monuments of industry)—its fertile fields—its neat and cleanly towns—its painted houses, varnished furniture, and broad-based, thick-headed inhabitants, excite a variety of emotions, and those generally of a pleasant kind, in the mind of the traveller —but FRANCE, from the Pyrenees to the Rhine, from the Jura to the Atlantic, from Antibes to Calais, presents very few spots indeed, compared with her vast extent of surface, on which the eye can rest with either pleasure or admiration.* Her mountains are destitute of sublimity—her valleys of beauty. Her roads are still, in most places, and at the best, but narrow, rude, and rugged chaussées, bordered, on each side, with mud in Winter, and sand in Summer; less calculated to 'speed the soft intercourse' among her inhabitants, than to demolish the springs of carriages, and dislocate the joints of travellers—designed, apparently, to check very effectually, the 'march of intellect,' by causing a concussion of the brain at every step! Her fields, though fertile, are fenceless, and slovenly cultivated, presenting a bald and frigid aspect. Her VINEYARDS, even in the Bordelais, along the smiling borders of the Garonne, resemble plantations of turnips when compared with those on the romantic banks of the Rhine, the sloping glades of Italy, or the upland scenes of Madeira. Her gentlemen's country-seats are in Paris; and their chauteaus are—in ruins—

'With nettles skirted and with moss o'ergrown.'

Her horses are rough, ugly, pot-bellied, ill-tempered, sour-countenanced, hard-working animals—the harness never cleaned or greased from the moment of its first construction till its final dissolution by winds and rains—her stage-waggons, y'cleped 'Diligences,' are loco-motive prisons or pontons, in which the traveller is pressed, pounded, and, what is worse than all, poisoned with mephitic gases and noxious exhalations evolved from above, *below*, and around.† Her provincial villages, towns, and even cities, are emblems of dulness:—long, narrow streets, with solitary lamps suspended at mournful distances in the middle, as if to point out the kennel that runs in the centre below, fraught with every kind of filth—HOUSES as if they had been shaken in a bag, and then jumbled together without regard to order, architecture, or any kind of regularity—tawdry painted exteriors, and cheerless, gloomy interiors—FLOORS without carpets, and hearths without grates—windows admitting as much air as light—fires without heat; easily kindled, rapidly consumed, and dearly paid for!—bell-ropes without bells, and servants without attendance—tables covered with a profusion of 'dishes tortured from their native taste,' and terrible to think of, much more to swallow! —vegetables drowned in oil or butter for the third or fourth course, and, after the Englishman has made a wretched dinner, like a cannibal—wine like vinegar

* "Even John Bell, from novelty and non-acquaintance with other countries, has launched out in extravagant praises of 'fair and fertile France.' His description of the scenery between Paris and Lyons is a caricature that will be very gratifying to Frenchmen—but it is a false picture. Excepting the banks of the Saone, between Macon and Lyons, the country is any thing but interesting. The spirited authoress of 'Rome in the 19th Century,' has drawn a more accurate picture, when she tells us that—'France is the most unpicturesque country in Europe. It is every where bounded by beauty, (the Alps, the Pyrenees, the Jura Mountains, &c.) but the country these grand boundaries inclose is remarkably devoid of beauty and interest. *It is a dull picture set in a magnificent frame.*'" Vol. I. p. 36.

† "It is very seldom that we meet with a foreigner, and especially a Frenchman, who has not a 'smoky chimney.' What with garlic dishes, rancid oils, everlasting cigars, and total inattention to mouth-washing, our Continental fellow-*voyageurs* are any thing but agreeable companions in a closed up diligence during the night!"

in the land of grapes!!—lastly, the BILL, (for I speak of hotels) a never-failing dessert, and often as *griping* as the wine, is modestly and conscientiously charged double, or nearly so, to the unfortunate ANGLOIS, who has not eaten a tithe of what his voracious Gallic messmates have consumed and pocketed!" 36.

### V. GENEVA.

"GENEVA itself is singularly well situated for health, cleanliness, and many of the mechanical arts, independently of the romantic and beautiful scenery surrounding it. A small island having split a magnificent river into two streams, immediately as it issues from one of the finest lakes in Europe, the town is thrown across this island and occupies the four opposite banks. Four level bridges maintain an easy communication between all parts of the town; and, as the houses project on piles over the river, the stream runs with a rapid course, not only through, but under a considerable portion of the streets and houses. Advantage is taken of this peculiarity of situation to abridge labour and save expense. It is not less curious than delightful to see the blue and 'arrowy Rhone' leap joyous through the streets of Geneva, ever ready and willing to lend its powerful aid to industry. It grinds their corn, washes their clothes, spins their cotton, cards their wool, turns their lathes—and, in short, is to the inhabitants a gigantic steam-engine, of inexhaustible power, voluntarily and gratuitously supplied by a thousand glaciers and ten thousand mountain streams." 47.

### VI. SION. CRETINISM.

"We are now in the centre of the Vallais—the head-quarters of goitre and cretinism. There are few portions of the earth's surface, in these temperate climes, better calculated for the deterioration, if not the destruction of life, than the Valley of the Rhone. It is bounded on each side by steep mountains, four or five thousand feet in height—and the intermediate ground contains all the elements that are found to operate against human health. The valley consists, in some places, of a rich, flat, alluvial earth, covered with corn, fruit trees, and gardens—in others, it presents swamps and meadows—then, again, jungle and woods—vineyards—pine forests, &c. while brawling brooks intersect it in all directions, and often inundate it, in their precipitous course from the mountains to the Rhone, which runs through its centre. Were this valley beneath a tropical sun, it would be the seat of pestilence and death. As it is, the air must necessarily be bad; for the high ridges of mountains, which rise like walls on the north and south sides, prevent a free ventilation, while, in Summer, a powerful sun beats down into the valley, rendering it a complete focus of heat, and extricating from vegetation and humidity a prodigious quantity of malaria. In Winter, the high southern ridge shuts out the rays of a feeble sun, except for a few hours in the middle of the day—so that the atmosphere is not sufficiently agitated at any season of the year. To this must be added, the badness of the waters which, along the banks of the upper Rhone, are superlatively disgusting.

As the Vallais is the land of cretinism, so is SION the capital of that humiliating picture of humanity! There are but few travellers who take the trouble to examine SION philosophically, and make themselves acquainted with the state of its wretched inhabitants. I explored this town with great attention, traversing its streets in every direction; and I can safely aver that, in no part of the world, not even excepting the Jews' quarter in Rome, or the polluted back lanes of ITRI and FONDI, in the kingdom of Naples, have I seen such intense filth! With the exception of two or three streets, the others present nothing on their surface but a nameless mass of vegeto-animal corruption, which, in all well-regulated towns, is consigned to pits, or carried away by scavengers. The alleys are narrow; and the houses are constructed as if they were designed for the dungeons of malefactors, rather than the abodes of men at liberty.

Goitre, on such a scale as we see it in the Vallais, is bad enough; but CRETINISM is a cure for the pride of man, and may here be studied by the philosopher and physician on a large scale, and in its most frightful colours. This dreadful deformity of body and mind is not confined to the Alps. It is seen among the Pyrenees—the valleys of the Tyrol—and the mountains of China and Tartary. Nearly 200 years have elapsed since it was noticed by Plater, in the spot where I am now viewing it; but Saussure was the first who accurately described this terrible degeneracy of the human species. From common bronchocele, and a state of body and mind bordering on health, down to a complete destitution of intelligence and sensibility—in short, to an existence purely vegetative, Cretins present an infinite variety of intermediate grades, filling up these wide extremes. In general, but not invariably, goitre is an attendant on cretinism. The stature is seldom more than from four to five feet, often much less—the head is deformed in shape, and too large in proportion to the body—the skin is yellow, cadaverous, or of a mahogany colour, wrinkled, sometimes of an unearthly pallor, with unsightly eruptions—the flesh is soft and flabby—the tongue is large, and often hanging out of the mouth—the eyelids thick—the eyes red, prominent, watery, and frequently squinting—the countenance void of all expression, except that of idiotism or lasciviousness—the nose flat—the mouth large, gaping, slavering—the lower jaw elongated—the belly pendulous—the limbs crooked, short, and so distorted as to prevent any thing but a waddling progression—the external senses often imperfect, and the Cretin deaf and dumb—the tout ensemble of this hideous abortion of Nature presenting the traits of premature old age! Such is the disgusting physical exterior of the apparently wretched, but perhaps comparatively happy, Cretin!" 57.

### VII. A NIGHT ON THE SIMPLON.

"The descent from the barrier to the village of the Simplon winds between wild, barren, and snow-clad heights—and the traveller is not sorry to ascend the dirty, cold, and stony stairs of the HOTEL DE LA POSTE into a dreary 'SALLE A MANGER,' where a stinking German stove, with its murky and sudatory atmosphere, is a miserable substitute for the blazing faggots of France, or the powerful radiation of light and heat from an English fire-side! Invalids should not stop here; but those who are in tolerable health should take two days to the Simplon, sleeping in this eagle's nest, in order to feel the contrast between the mountain air of the Alps, and the mephitic atmosphere of the Vallais. In a small apartment, ten or twelve feet square, I was fortunate enough to find a chimney, and took good care to kindle a cheerful fire. I had walked almost the whole way up the Simplon—made a hearty dinner—and taken my bottle of *mountain* wine. The crackling of the pine faggots, the murmuring of the tapering flame, the genial warmth of the ungrated hearth, the circumscribed dimensions of my little chamber, the howling winds, descending in fitful blasts from the Schonhorn, the Fletschorn, and the hundred surrounding glaciers, shivering the broken panes and disjointed frames of my little window, disturbed not, but rather aided, an hour's rumination, with all its discursive ranges among the fields of fancy, memory, and imagination, till a sleep, too deep for dreams, and such as monarchs have vainly sighed for, with all the opiates of wealth, power, and pleasure, sealed my senses in seven sweet hours of heavenly and restorative oblivion." 63.

### VIII. PELLAGRA OF LOMBARDY.

"A phenomenon resulting from the physical operation of climate on the human race, and which is equally curious and melancholy to contemplate, may be seen on a large scale in the great hospital of Milan—the PELLAGRA of the Lombardo-Venetian plains. Those who have not courage to view it in the living body, may form a tolerable idea of its external characters from some excellent representations in wax, at the Museum of the University of Bologna.

This horrible malady, or complication of maladies, has only been observed during the last 60 or 80 years, and is rapidly increasing. The proportion of cases in the hospital is very considerable.* It begins by an erysipelatous eruption on the skin, which breaks out in the Spring, continues till the Autumn, and disappears in the Winter—chiefly affecting those parts of the surface which are habitually exposed to the sun or the air. This cutaneous symbol of an internal disorder is accompanied or preceded by remarkable debility, lassitude, melancholy, moroseness—hypochondriacism—and not seldom a strong propensity to suicide. Year rolls on after year, and the cutaneous eruption, as well as the general disorders, become more and more aggravated, with shorter and shorter intervals in the Winter. At length the surface ceases to clear itself, and becomes permanently enveloped in a thick, livid, leprous crust, somewhat resembling the dried and black skin of a fish ! By this time, the vital powers are reduced to a very low ebb—and not seldom the intellectual functions. The miserable victim of this dreadful pellagra loses the use of his limbs, more particularly of the inferior extremities—is tormented with violent colick, head-ache, nausea, flatulence, and heartburn—the appetite being sometimes nul, at others voracious. The countenance becomes sombre and melancholy, or totally void of expression—the breath fetid—the teeth rotten—the inside of the mouth ulcerated—the mucous membrane highly irritable, and diarrhœa is a common accompaniment of the other disastrous train of miseries. But the most distressing phenomenon of all, is a sense of burning heat in the head and along the spine, from whence it radiates to various other parts of the body, but more especially to the palms of the hands and soles of the feet — tormenting the wretched victim day and night, and depriving him completely of sleep ! He frequently feels as if an electric spark darted from the brain, and flew to the eyeballs, the ears, and the nostrils, burning and consuming those parts. To these severe afflictions of the body are often added strange hallucinations of the mind. The victim of pellagra fancies that he hears the incessant noise of mill-stones grinding near him—of hammers resounding on anvils—of bells ringing—or the discordant cries of various animals ! The disease when advanced, takes the form of many other maladies, as tetanus, convulsions, epilepsy, dropsy, mania, and marasmus†—the patient ceasing at last to exist and to suffer, when reduced to the state and appearance of a mummy. It is by no means uncommon—who can say it is wonderful—that the wretched being abbreviates the term of his afflictions, and anticipates the too tardy hand of death in a paroxysm of suicidal mania ! It is remarkable that this tendency to self-destruction very often assumes the form of a desire to consummate that last act of the tragedy by drowning—so much so that STRAMBI, a writer on the pellagra, has given it the name of HYDROMANIA, when this propensity exists." 76.

### IX. A NIGHT ON THE APENNINES.

" Choice induced us to spend a night ' above the storm's career,' in the village of the Simplon. Necessity—or rather the GOD of LOVE, compelled us to sleep in a ' house of ill fame,' on the summit of the Apennines. The cavalcade of a marriage in high life—the betrothed Princess of Naples on her way to wedlock with her uncle, Ferdinand the embroiderer—stopped us full an hour between Pietra Mala and Caviliajo, obliging us to sleep at the latter place, a solitary inn in the centre of these mountains—' the scene of one of those deep-laid

---

* " It has been supposed that a sixth or seventh of the population is affected with pellagra, in those parts of the country where it is most prevalent."

† " It is on this account that we see written over the heads of the beds in Milan Hospital, the various diseases to which pellagra forms the adjective, as atrophia pellagrina, phthisis pellagrina, hydrops pellagrinus, paralysis pellagrina, mania pellagrina, &c."

confederacies for plunder and assassination, of which Italy has always been a prolific theatre.' We had the pleasure of reading in Forsyth, that, from this same inn, 'travellers daily disappeared, and could never be traced by their spoils.' Two of his acquaintances escaped by stratagem; and, not long afterwards, the banditti were surprised while feasting at the parsonage in the neighbourhood, when the horrible mystery was revealed.

'It was the law of this society to murder all the passengers they stopped—to kill and bury the horses, burn the carriages and baggage, rescuing only the money, jewels, and watches. BIONDI, the curate, was their captain—the MISTRESS of the INN was their accomplice, who sent him notice of every traveller that lodged at her house.'

Notwithstanding this astounding intelligence, we supped very comfortably, and I retired to my chamber, which was in the back of the house, over the stables—the window being without fastenings, and a pile of stones reaching up to within two feet of the window-sill, from a dreary and suspicious wood, offering a most tempting facility to any of BIONDI's gang who might wish to pay me a nocturnal visit. In despite of this appalling history and these ominous phenomena—nay, in spite of a tremendous storm of 'thunder, lightning, and of rain,' which demolished the few remaining panes of glass in my chamber window, I slept as soundly, and I believe as safely, as I should have done in 'Modern Babylon.' A journey from one end of Italy to the other—sometimes with tempting equipage—sometimes as a solitary, unarmed, and defenceless rambler, has convinced me that, with common prudence and good humour, a traveller is as safe in this land of banditti, as in any part of the British dominions. An Italian will outwit you—or, if you please, cheat you, in every possible way—but he will not murder you—pillage you—or steal from you, if you leave your baggage open in the court of the inn where you sleep." 86.

### X. CLIMATE OF FLORENCE.

"Such is the far-famed Arno, along the banks of which the public promenades are constructed, and take, on both sides of the river, as well here as at Pisa, the name of LUNG' ARNO—signifying, on the right bank, LUNG-WARMER—on the left, LUNG-HARMER. The span of the Trinita or Carraja Bridge makes all the difference between Summer and Winter in Florence. The LUNG' ARNO, on the North side of the river, being sheltered by the city from the tramontane winds, and open to the sun, is warm, or even hot—while, at the same moment, Schneider's side being exposed to the Apennine blast, and excluded from the solar beams, is chilling cold. And yet the warm side of the Arno is the more dangerous of the two for the sensitive invalid. Thus, while pacing the promenade between the two bridges above-mentioned, the wind being northerly, the temperature will be felt very high, so as readily to bring out perspiration; but the instant we come abreast of any of the streets at right angles, such as the Piazza St. Trinita, or the Vigna Nuova, we are stricken by an icy current of air, the more injurious, from the open state of the pores and the sudden transition of the temperature. On the other side of the Arno it is permanently more cold, and, when the Sirocco prevails, we are exposed to currents of that debilitating and suffocating wind at the crossings of streets—but these are not dangerous. From whatever point of the compass, however, the breezes blow—along whatever street they sweep, even in this pride of Italian cities—they carry on their wings, not 'airs from Heaven,' but 'blasts from Hell,' saturated with reeking vapours from—all 'UNUTTERABLE THINGS.' Mr. Eustace tells us, indeed, that Florence is 'airy, *clean*, and sometimes rising towards grandeur.' I deny the second assertion, and I appeal to ocular demonstration, not merely in obscure streets, but throughout every piazza and square in that great capital. Let the traveller walk, for instance, through the PIAZZA ST. MARIA NOVELLA (the largest in Florence) in the middle of day, and let him halt before the obelisk in its centre. He will there see what I shall not describe. If not satisfied, let him repair to the PIAZZA DEL DUOMO itself, and there contemplate the *Pagan sacrifices* that are offered up along its sacred walls in broad noon-day! Nay, I assert that the 'gates of Paradise,' as Michael Angelo styled the portals of the Baptistery, are unsafe to enter, unless we afterwards have recourse to the 'holy water' in the FONT, to purify our bodies as well as sanctify our souls. Look at the PALAZZO PITTI, the residence of royalty. It is very gloomy and very grand. The bayonet keeps its walls undefiled. But turn down into any one of the streets that lead from that splendid palace towards the banks of the Arno, and the *unaccustomed* eye will revolt from the accumulations of filth and corruption that every where present themselves! Such are the scenes wherein the young gentry and nobility of England are to form their taste, polish their manners, refine their senses, cultivate their understandings—and *finish* their education!" 93

### XI. THE VENUS DE MEDICIS.

"The TRIBUNE (the sanctum sanctorum of the gallery) is wisely reserved by the custodes for the last exhibition in their Sysiphean occupation. I entered it the first day, without knowing where I was going. The VENUS DE MEDICIS instantly told me I was in the presence of beauty personified. Her averted look certainly indicates, according to my impression, some degree of shame, or even denial. When we advance and turn to the right, so as to command her countenance, I fancied that I could perceive a triumphant if not a sarcastic smile, playing on features that are mellowed rather than faded by TIME. The position of the hands is more artful than honest—pointing to, rather than concealing what female modesty has veiled from observation.

'Ipsa Venus pubem, quoties velamina ponit,
Protegitur læva semi-reductâ manu.'

This charmer has been called the MADONNA DELLA CONFORTA, for ladies of low stature, and a certain or rather *uncertain* age—being herself under five feet in height, and some three centuries on the wrong side of the Christian æra, in years. Time has embrowned her complexion; but her FIGURE remains the admiration of the world. It may appear somewhat paradoxical to say that, the whole form is perfection, though many of the principal parts are faulty. The critics have determined, among other blemishes, that the head and the hips are too small—that the nose and the hands are too large—that the fingers are like marlin-spikes, and have only one joint among ten of them. The diminutive head would not have been of much consequence, had not the phrenologists, with their callipers, ascertained that the owner of the head was an idiot! Well! This would not much diminish the number of her admirers. Praxiteles or Cleomenes was not so silly as to give VENUS as much brains as MINERVA. It is not necessary—it is not desirable, that a BEAUTY should be a BLUE-STOCKING. What did SAPPHO gain by her towering intellect and tender lyrics? Not a husband certainly, unless under the Ægean wave!

Say lovely youth that do'st my heart command,
Can Phaon's eyes forget his Sappho's hand?

No truly! Phaon remembered her *head* as well as her hand, and kept at as respectful a distance from the tenth Muse as a butterfly BEAU does from a literary BELLE, of the present times.

But then the pelvis is not so broad, nor the rump so prodigious, as among the Hottentot Venuses of our own day. This is true. The poor VENUS DE MEDICIS had not a Parisian milliner to exaggerate the *deformities* of Nature. Yes, the *deformities* of Nature! Unless the critic is prepared to maintain that black and white are the same colour—that great and small dimensions are of the same admeasurement, I do assert that Nature, for wise purposes, has imposed a tribute of deformity on the beauty of the female sex, which, it is the duty of the sculptor and painter to diminish, in beau-idealism, rather than exaggerate. Praxiteles, Cleomenes, or whoever it was that sculptured the Medicean Venus, acted judiciously in diminishing the size of the female pelvis, the dimensions of which, however useful for the perpetuation of the human race, can never be pleasing to the eye of the spectator. I could illustrate this position, and demonstrate that NATURE does not always study symmetry and proportion in her formations or operations; but I think it unnecessary. The most beautiful female figure, in youth and in health, differs totally in the course of a few months. Poets, painters, and sculptors, have taken care to delineate but one side of the portrait." 106.

### XII. EFFECTS OF ITALIAN MALARIA.

"A glance at the inhabitants of malarious countries or districts must convince even the most superficial observer, that the range of disorders produced by the poison of malaria, is very extensive. The jaundiced complexion, the tumid abdomen, the stunted growth, the stupid countenance—the shortened life, attest that habitual exposure to malaria saps the energy of every bodily and mental function, and drags its victims to an early grave. A moment's reflexion must shew us that FEVER and AGUE, two of the most prominent features of the malarious influence, are as a drop of water in the ocean, when compared with the other less obtrusive, but more dangerous maladies that silently but effectually disorganize the vital structures of the human fabric, under the operation of this deleterious and invisible poison. Yet the English traveller or sojourner in Italy knows little, if any thing, respecting these slow and masked underminings of his health, and thinks, if he escapes the malaria fever of July and August, he has nothing more to dread, but every thing to enjoy, throughout the year. Fatal mistake! The foundation of chronic maladies, that render life miserable for years, is every Summer laid in hundreds of our countrymen, who wander about beneath the azure skies of Italy. They bring home with them a poison circulating in their veins, which ultimately tells on the constitution, and assumes all the forms of Proteus, harrassing its victim with a thousand anomalous and indescribable feelings of wretchedness, inexplicable alike to himself and his physician. It is the attribute, the character, of all malarious disorders to be slow in their development, when the poison is inhaled in a dilute state, or only for a short time. Many of our soldiers did not feel the effects of the Walcheren malaria till months, or even years, after that fatal expedition. So our countrymen in India often go on for years in tolerable health, after exposure to a malaria, before the noxious agent shews itself in the disturbance of certain functions of the body. The same thing is seen even in England, though on a smaller scale. Those who inhabit marshy or damp situations become, sooner or later, affected with some of the Proteiform maladies engendered by malaria, though they are seldom understood, unless they happen to take on a regular aguish character.

Two causes have a marked influence in deranging the biliary and digestive organs—solar heat and terrestrial exhalations. Either is equal to the production of the effect; but, when combined, the agency is most potent. Thus, in India and other tropical climates, when a high range of temperature combines with marsh miasmata, liver and bowel-complaints are sure to result. And, under the most favorable circumstances, although hepatitis or dysentery may be evaded, the organs of digestion are sure to suffer in the end; and the melancholy catalogue of dyspeptic, bilious, and nervous complaints is the portion of the tropical sojourner. Now Italy, in Summer and early Autumn, is nearly as hot as the East or West Indies, and is the very throne of malaria. She has also the additional disadvantages of the sirocco and tramontane winds—or, in other words, vicissitudes of temperature, great and sudden, beyond any thing which we witness even under the Equator. What are the consequences? Malarious fevers;—or, if these are escaped, the foundation of chronic malarious disorders is laid, an ample provision for future misery and suffering!" 125.

### XIII. COLISEUM.

"Of all the monuments that now exist to attest the decline and fall of the Roman empire, this is the most stupendous;—and, could it be dissociated, in the mind, from the causes which gave it birth, or the cold-blooded hideous barbarities which it exhibited, it would be the most majestic, even in its ruins. But the springs of action are more philosophic objects of contemplation than the mere machinery by which these are brought into operation. In the early years of a state, as in those of an individual, the sensibilities, though keen, respond only to natural impressions. But as time rolls on, as wealth accumulates, as luxury prevails, and as virtue decays, the sensibilities become not only blunted, but perverted—wholesome stimuli cease to call forth the usual, or at all events, the desired excitement—and then nature is outraged in every possible way. Such was the condition of the Romans, when the manly, or at least the innocent, contests of the circus, and the fictitious sorrows of the stage became insipid—and yon gigantic structure rose, arch over arch, and order over order, Titan-like, to scale the heavens; or, rather, to usurp the privileges of the gods, in receiving the incense of slaughtered victims—in breathing the odour of human gore, jetting in crimson fountains from a thousand pierced and palpitating hearts.

To feast their eyes on the mangled and quivering members—on the reeking entrails of man and animals—to view, with exquisite delight, the murderous conflicts of the ensanguined arena, hither flowed daily the impetuous tide of human existence, the lords of the creation, the venerated, the god-like Romans! Here took their allotted seats, the sceptered prince and laurelled consul—the war-like knight and solemn senator—the haughty patrician, and factious tribune—the vestal virgin, and stately matron—the tuneful bard and grave philosopher. These and countless multitudes of Roman citizens and Roman rabble, rushed daily to yon gorgeous structure—all for the sake of that EXCITEMENT which simple or innocent pleasures could no longer elicit!

Yes! and when the wounded gladiator fell before the superior force or fortune of his fierce antagonist, and sued for life—when the victor poised in air his gory falchion, and looked for the signal of mercy or murder—these polished Romans—the fair-sex themselves, vestals maidens and matrons, held up their hands for BLOOD; nor would they forego the poignant pleasure of seeing the reeking steel plunged into the vitals of a fellow-creature!* Such was yon colossal slaughter-house, where every ferocious animal that roamed the wilds or haunted the rivers of Asia, Africa, and Europe, was conducted to view, as well as to encounter, with horror and astonishment, the still more ferocious animal—MAN.†

---

* "'Two aqueducts were scarcely sufficient to wash off the human blood which a few hours *sport* shed in this imperial shambles. Twice in one day came the Senators and Matrons of Rome to the butchery: a virgin always gave the signal for slaughter, and when glutted with blood-shed, those ladies sat down in the wet and streaming arenæ to a luxurious supper.' Who would expect that Cicero should not only defend, but warmly commend gladiatorship: 'Oculis nulla poterat esse fortior contra dolorem et mortem disciplina.' If this diabolical insensibility to scenes of blood and murder was expected to rekindle the valour of the degenerate Romans, the expectation was most woefully disappointed! The horrible and debasing inference of Cicero, indeed, is negatived by the examples of ancient Greece and modern Europe. Compare the heroic retreat of the ten thousand Greeks with the shameful flight of Julien's Roman legions from the banks of the Euphrates."

† The licentious and blood-thirsty Romans did not always enjoy these sights with impunity. When the Emperor Probus was preparing for his triumph, nearly 300 years after the birth of Christ, fourscore desperate gladiators, out of

Erected by a Pagan—purged of its inhuman rites by a Priest—and propped in old age by a Pope—the Coliseum shadows out some faint emblematical picture of Rome itself. It was once the stormy theatre of bloody deeds—it is now the peaceful asylum of holy crosses. Part of it still stands erect, or renovated; part of it totters over its base; but the greater part has vanished. Eloquent in its silence, populous in its solitude, majestic in its adversity, admired in its decay, the ruins of the Coliseum, like the remains of Rome, excite the curiosity of the antiquary—the ruminations of the moralist—the zeal of the Catholic—the admiration of the architect—the sigh of the philanthropist—the sneer of the cynic—the humiliation of the philosopher—and the astonishment of all." 144.

### XIV. ITALIAN CHARACTERISTICS.

"The external physiognomy of Italy, as well as of her great cities—and even of her inhabitants, presents more prominent features and singular contrasts than any other country or people in the world. Bernardine de St. Pierre informs us that all contrasts produce harmonies—and hence, perhaps, it is, that Italy is the land of music and of song. There is poetry—or the materials of poetry, in every thing which meets the eye between the Alps and Mount Ætna. Her skies are azure and her hills are green—the sun-beams are ardent, the moonbeams mellow, the stars brilliant—the breezes are alternately delicious and malarious—iced by the Alps, or ignited by the Sirocco—her mountains are lofty, and her streamlets are clear—her rivers are rapid, and her lakes are smooth—her shores are laved by tranquil seas, her hills are shook by hidden fires—the country is rich, and the people are poor—the fields are fertile, while their cultivators are squalid and unhealthy—men and women sew the seed; but saints and angels reap the harvest—the vines are graceful, the grapes luscious; but the wine is too often sour—the roads are magnificent, while the inns are wretched—the country swarms with priests, but is destitute of religion—teems with redundant population where celibacy is the CARDINAL VIRTUE—glitters with gems and precious stones in the midst of penury and starvation—exhibits despots on the plains, and bandits in the mountains—abounds in all the materials of wealth and power, but possesses few flourishing manufactories, except those of monks, music, and maccaroni. In fine—the nobility is sunk in sloth, the church in plethora, the populace in pauperism!" 171.

### XV. PASSAGE OF THE PONTINE FENS.

"'Et quos pestifera Pomptini uligine Campi.'

"The brigand-looking groups of Velletri proved as harmless as the mountaineers of Tuscany—we safely descended to the marshes—and were soon in sight of the TORRE DE TRÉ PONTI, where we observed, at some distance, the squalid caliban borderers collecting wild beasts from the fens, and beating as well as swearing them into office, as post-horses, for our accommodation!

Four of these savage and unseemly creatures being pinioned to the large, and two to the small carriage, away they flew—kicking, flinging, plunging, and snorting—curvetting in fitful and fearful sallies from side to side of the road—one moment within an inch of dashing us to pieces against the trunk of an elm or a poplar—the next, within an ace of hurling us headlong over a perpendicular

---

600 who were reserved for the inhuman sports of the Coliseum, disdaining to shed their blood for the amusement of the populace, broke from the place of their confinement, and filled the streets of Rome with slaughter and confusion. They were overcome at last; but not before they avenged their fraternity by torrents of blood in the Eternal City.

bank into the yawning canal below—keeping us in perpetual, and not the most agreeable suspense, between a broken neck and a watery grave! Thus we darted across the Pontine Fens, with little less velocity than that of an arrow from a bow—traversing a space of 28 miles (nearly the distance between London and Chatham) in two hours and forty minutes—a *journey* which, in the Augustan age, and in the pride of the VIA APPIA, occupied Horace during sixteen tedious hours, while listening to the croaking of frogs, the brawlings of boat-men, the maledictions of muleteers—the buzzing of gnats—

Mali culices ranæque palustres—

and what was far worse, while submitting to the depredations on personal property inflicted by those douaniers of antiquity, the bugs, the fleas—and a certain animal—

Quod versu dicere non est." 194.

### XVI. DRIVE TO POMPEII.

"If a stranger were to arrive at Naples, by sea, and that for the first time, in the month of November or December, he would be apt to form a very erroneous idea of the climate, according to the point from which the wind blew. If it came from the SOUTH, he would be inclined to think that there was little difference between Naples and the black-hole of Calcutta. If from the NORTH-EAST, he would begin to doubt whether he had not sailed in the wrong direction, and made the Gulf of Finland, instead of the Gulf of Salerno. If a gentle North-West zephyr skimmed the surface of the deep and wooed the shores of Baiæ, he might be tempted to think that he had got into the gardens of the Hesperides, or the isles of Atlantis, so green is vegetation, so balmy the air, so mellow the sun-beams, and so azure the skies!

YESTERDAY, the SIROCCO—'Auster's sultry breath'—steamed over Naples, depressing the animal spirits and the vital energies to the lowest ebb. It is impossible to convey in words any adequate idea of the sedative effects of this wind on mind as well as body. I tried to respire in freedom on the roof of the Vittoria—on the Chiaja—the Mole—the Chiatomone; but found no relief from the nervous depression and muscular languor induced by this mephitic composition of rarified air and aqueous exhalation. I hired a calessino and drove round the promontory of Posilipo—and afterwards ascending to the airy castle of ST. ELMO, wandered through the beautiful church of ST. MARTINO—but all in vain! From lassitude of body and dejection of mind there was no escape, while this accursed blast prevailed.

TO-DAY, started at sunrise, in an open barouche, for POMPEII, under the chilling influence of a TRAMONTANE, or North-easter, that came down in piercing gusts from the Apennines, more cutting and keen than the winds that sweep along the Winter snows of Siberia. In passing through PORTICI, I could scarcely help envying as well as pitying the LAZZARONI, stowed in rows, like sailors' hammocks, along the sunny sides of the streets, sheltered from the blast, and basking in the rays of a glorious luminary.

As the carriage rolled rapidly over the volcanic grave of HERCULANEUM, hollow murmurs echoed from the chambers of the dead beneath; while fancy assimilated these melancholy sounds with the dying groans of its entombed inhabitants, when the terrific surge of boiling lava curled for an instant against the ramparts, and then swept, with relentless fury, over the devoted city! No sight—no idea is so agonizing to the human mind, as that of protracted torture and lingering death. Fortunately for the Herculaneans, their sufferings were momentary, and instant destruction released them from the horrors of the scene! The nature of the fatal torrent which inhumed Herculaneum, and filled every crevice with solid stone, will probably prevent its ever being excavated." 207.

### XVII. STATE OF MORALITY AMONG THE POMPEIANS.

"That a lady's work-box or flower-stand should be supported by pedestals of curious or elegant workmanship, no one can object; but if we find these pedestals moulded or carved into the most disgusting and obscene figures which a depraved imagination could invent, are we not authorized to conclude that the female mind was corrupt and rotten to the very core? Those who have examined the penetralia of the MUSEO BORBONICO—nay, those who look at the drawings made by order of the CHANOINE JORIO himself, must confess that this picture is not overcharged! The same depravity is too often seen to pervade every kind of female ornament—the necklace, the ear-rings, the bracelets, the amulets—every object, in short, on which the female eye was accustomed to repose!

But however humiliating is the picture of female *indelicacy* (not to give it a coarser name) that of the male sex very far surpassed it, and ran into the grossest BESTIALITY. Will it be believed that a MAN, before his own death, or his FRIENDS, after that event, should have employed sculptors for months, or years, in *decorating* the marble sarcophagus in which the lifeless corpse was to repose, with the grossest emblems and representations of revolting obscenities and crimes! Such, however, is the fact—and this fact alone is damning proof enough of the state of depraved feeling in which the Roman mind was sunk at the commencement of the Christian æra." 214.

### XVIII. PASSAGE OF THE PONTINE MARSHES BY NIGHT.

"We started from Terracina, a little before sunset, in a carriage very badly calculated for four, but compelled by the villainous courier of the Pope (for which I hope he has never received absolution) to hold an additional passenger, in the shape (if shape he had) of his own pot-bellied son, besides baggage and luggage enough to load a caravan. Nothing but the philosophy of observing the Pontine Marshes at night, could have induced me to bear, with any degree of patience, the infernal breath of the father and his urchin, between whom I voluntarily placed myself to give the invalids all the accommodation which their health and sufferings required. But patience has its bounds, and at the end of the first stage I got on the outside of the coach, rather to breathe the deleterious gases emitted from the fens, than inhale the mephitic airs generated within this infernal cauldron. The atmosphere was still as the grave—the moon shone faintly through a halo of fogs—and a dense vapour rose in all directions around us, emitting the most strange and sickly odour which I ever experienced on any part of the earth's surface. Under other and ordinary circumstances, I should have felt some alarm at thus exposing myself to the full influence of nocturnal emanations from the deadly marshes over which we were passing; but a consciousness of the life which I had led for three months, inspired me with complete contempt for any morbific influence which air or earth could direct against me. I crossed the fens in this philosophic mood, while the courier of St. Peter kept the windows of the coach closely shut against the dangerous malaria of the night. I would not advise others to imitate this rash conduct on my part. Many have paid dearly for their curiosity—and myself among the rest—if not on this, on various other occasions.

———— Video meliora proboque
Deteriora sequor!" 219.

### XIX. PASS OF THE BRACCO.

"Our afternoon's journey, from Borghetto to Sestri, was over the BRACCO, an Apennine pass, little inferior to the Simplon in height, in distance, and in majestic scenery. The ascent is 12 miles, and the descent on the other side is eight miles. The village of Mattarano, like the village of the Simplon, is near the summit—and four miles beyond this forlorn cluster of human habitations, and at the highest point of elevation, is a place of refuge in case of snow-storms. The road, which is excellent, winds along terrific precipices at one time, and, at another, is overhung by the most horrifying cliffs and crags on which human eye ever ventured to gaze. Any one of the hundred jutting masses of marble that hang suspended, a thousand feet high, over the traveller on this route, would, if disrupted, overwhelm a whole city! The aspect of the mountains all around is wild and savage beyond description, or even imagination—and the loneliness of this desert, (for scarcely a human creature met our eye) for twelve or fifteen miles, adds to the solitude, the silence, the gloom—and yet to the sublimity of the scene! Painters, poets, and romance-writers would find ample materials for contemplation and study between Pisa and Nice—and the Mountain of Bracco would furnish them with a scene of the terrific at any time. And not of the terrific only! for it is from the highest and wildest part of its summit that, all at once, the Bay of Rapallo, little inferior to that of Spezzia, with all its romantic shores, bursts on the view, awaking the most pleasant sensations by contrast with the gloomy horrors of the mountain—and by giving assurance that we have only a rapid descent of eight miles to the end of our journey, and comfortable refreshment in an excellent inn."* 232.

### XX. CHARACTERISTICS OF THE NEW ROAD FROM GENOA TO NICE.

"The route from Genoa to Nice is equally interesting, as that between Pisa and Genoa, though exhibiting a very different cast of scenery. Were I to attempt a short graphic sketch of its more prominent and characteristic features, to which nothing but the pencil of a Linton, however, can do justice, I would say that, the broad, the blue, the boundless, and the tideless Mediterranean, here chafing against the wave-worn rocks, there murmuring on the golden sands, always in view, always in close proximity, forms the grand and glorious feature of the prospect, on one hand, to which the eye repeatedly turns for refreshment, and on which the imagination loves to roam, as well as to rest. On the other hand, and in magic contrast, Alps and Apennines rise in every variety of form, capped with everlasting snows, girdled with mighty forests, and based with perennial verdure. Between these majestic scenes, the towns and villages, white as Parian marble, are seen stretched in lengthened curves along the sinuosities of the bays; perched in irregular clusters on airy cliffs; or clinging, in fearful suspension, on the precipitous descents of the hills. The road now steals, in quietude and smoothness, along the very verge and level of the placid ocean—now creeps up the forbidding acclivity of a rugged steep, in slow and labouring zig-zags—winds along the furrowed brow of a lofty mountain—dives through the solid marble, and emerges on the edge of a giddy precipice, a thousand feet perpendicular above the murmuring surge, on one side; a thousand feet beneath overhanging and gigantic masses of rock on the other. Anon, the road strides, arch upon arch, over a frightful chasm, or impassable ravine — descends, by tortuous but gentle windings, the horrid steeps of a wild, a gloomy defile — and loses all trace of its existence on the broad and rugged bed of a mountain torrent." 242.

### XXI. CLIMATE OF ITALY.

"Italy, indeed, is very singularly situated in respect to climate. With its feet resting against the snow-clad Alps, and its head stretching towards the burning shore of Africa, it is alternately exposed to the suffocation of the sirocco, from the arid sands of Lybia, and the icy chill of the tramontane from

---

* "There can scarcely be imagined a more appropriate scene for robbery and assassination, than the pass of the Bracco. Travellers might here be shot or felled to the ground by hands unseen among the over-hanging crags; and when pillaged, their bodies might be hurled over precipices beyond all human search! Yet here are no guards—no patrole—and no more danger than between Dover and Canterbury!"

the Alps or the Apennines. The elevated ridge of mountains that bisects the whole of Italy longitudinally, operates powerfully in modifying her climate.

Against the summits of this rugged and lofty chain of Apennines the sea-breeze that has swept the Mediterranean or even the Atlantic Ocean, on one side, or the Adriatic on the other, strikes often with great violence; but is, on the whole, impeded in its course—more especially the lower strata of air—hence the stillness of the atmosphere so remarkable at ROME and many other parts of the western plains and valleys of Italy. This stillness is by no means advantageous, in point of salubrity, to a country where deleterious exhalations are hourly issuing from the soil in the Summer season, and which are dissipated by winds and concentrated by calms. Thus, then, this Apennine ridge affords no protection from the chilling blast of the Alps, or the enervating sirocco of Africa; while it diminishes the utility, by obstructing the current of the sea-breezes, from whatever point they may blow. But the Apennines themselves, when they annually resume their caps of snow, become the source of most piercing and cutting winds, more chilling than those from the Alps, on account of their greater proximity to the plains. The Apennine, therefore, is one of the agents which produce those excessive transitions of temperature, to which the atmosphere of Italy is subjected."

"From the relative situation, then, of the Alps, the Apennines, and the sands of Africa, it may be said that almost every breeze in Italy comes over a volcano or an iceberg—and, consequently, we are alternately scorched by the one and frozen by the other.

There is a vast difference between the *variability* of climate in England and in Italy. In England, the changes (barometrical, thermometrical, and hygrometrical) are very *frequent*, but they are also very *limited in their range*. In Italy, it is just the reverse—the transitions are *not* very frequent; but, when they do occur, the range is often most extensive. Now the *frequency* of alternations in England, and the moderate range of these alternations, are the very circumstances which render them comparatively innocuous. We have cloud and sunshine, heat and cold, winds and calms, drought and rain, twenty times in one day at home; but the British constitution becomes inured to them, and safely so, from the rapidity of their recurrence and the limitation of their range. Nay, this perpetual scene of atmospheric vicissitudes not only steels us against their effects, but proves an unceasing stimulus to activity of body and mind, and, consequently, to vigour of constitution."

"If, thermometrically speaking, we say that the Summer heat of the Italian valleys approaches the temperature of the tropics*—while the tramontane blast of Winter depresses the mercury as much as a Caledonian North-easter—we convey a very inadequate idea of the *feelings and the physical effects* occasioned by these opposite conditions of the atmosphere in Italy. I have alluded to this subject, under the head of Naples, and also on the journey from Genoa to Nice. The thermometer, in fact, is no index or criterion of our feelings under the influence of the SIROCCO and TRAMONTANE. The *former* appears to suspend, exhaust, or paralyze the nervous energy of the body, and the sensorial vigour of the mind; both of which fall prostrate beneath the flood of enervating steam engendered by the aerial current sweeping over burning sands and evaporating seas. The *latter*, or tramontane, comes down from the Alps or Apennines, with such a voracious appetite for caloric, that it sucks the vital heat from every pore—shrivels up the surface of the body—impels the tide of the circulation, with great violence, upon the internal organs—and endangers the lungs or whatever other structure happens to be weakest in the living machine."

* "Dr. Clark states the mean temperature of the Mediterranean generally, in the month of August at 80° Fahrenheit, which is very little less than the mean annual temperature of the Indian Ocean."

"The very circumstance, in short, which forms the charm, the attraction, the theme of praise in the Italian climate, is that which renders it dangerous, because deceitful—namely, the *long intervals of fine weather between vicissitudes of great magnitude.* This is the bane of Italy, whose brilliant suns and balmy zephyrs flatter only to betray. They first enervate the constitution; and, when the body is ripe for the impression of the TRAMONTANE, that ruthless blast descends from the mountains on its hapless victim, more fierce and destructive than the outlawed bandit on the unsuspecting traveller!

Italy boasts much of the dryness of her climate. In some places, as at Pisa, there falls as much rain as in Cornwall. In Rome, about one-third less of rain falls than at Penzance, and the number of rainy days is one-third less—being about 117 in the year. This is a poor counterbalance for the *steam* of the Sirocco, and the oppressive stillness of the Roman air. The fogs of England and its cloudy skies furnish constant themes of querulous complaint; but they would be rich treats in Italy, as defences against the torrents of liquid fire that pour down on her vales from a nearly vertical sun in Summer. As rains fall in Italy more seldom than in England, they make up for this infrequency, by precipitating themselves in cataracts, that form mountain torrents which overflow their banks, flood the plains, and saturate every inch of ground with humidity. The deluge over, a powerful sun bursts forth, and rapidly exhales into the air, not only the aqueous vapour from the soil, but the miasmata generated by the decomposition of all the vegetable and animal substances which the rains have destroyed, the floods carried down from the mountains, or the gutters swept out of the streets. If these exhalations rise into the air perfumed with the aroma of ten thousand odoriferous shrubs, breathing their balmy influence over the face of a smiling landscape, they are not the less, but the more dangerous on that account.

Northern strangers, and more especially INVALIDS, unaccustomed to an azure sky and a genial atmosphere in the depth of Winter, sally forth to enjoy the glorious sunshine or resplendent moonlight of Italy—and, like the Grecian shepherds—

——————— Exulting in the sight,
Eye the blue vault, and bless the cheerful light!

But they have, too often, reason to curse, in the sequel, the seductive climate of this classic soil, which mingles the poisonous miasma with the refreshing breeze, and thus conveys the germ of future maladies on the wings of fragrant Zephyrs." 262.

We had intended to exhibit a series of specimens from the third part of the work, on the medicinal and the moral influence of Italian skies and voluntary expatriation; but the extracts already introduced have run further than we expected, though compressed by means of the smallest type. We have given enough to enable the reader to form some idea of this little volume, the intention of which is to combine utility with amusement, on a journey in search of health; but more especially to put medical men, as well as their patients on their guard against incautious embarkations for a foreign climate in various diseases, which are more frequently exasperated than remedied by such procedures.

